# PolLimCrop, a global dataset of pollen limitation in crops

**DOI:** 10.1038/s41597-023-02797-6

**Published:** 2023-12-15

**Authors:** Catarina Siopa, Helena Castro, João Loureiro, Sílvia Castro

**Affiliations:** Centre for Functional Ecology, Associate Laboratory TERRA, Department of Life Sciences, University of Coimbra, Coimbra, Portugal

**Keywords:** Agroecology, Ecosystem services, Plant reproduction

## Abstract

Pollination is a crucial ecosystem service for maintaining plant communities and food production. 75% of the main crops depend on or benefit from pollination services provided by animal pollinators. However, when these services are insufficient and/or inefficient, crops experience pollen limitation with, often, lower associated yield, which may translate into economic losses. We constructed a global dataset that gathers studies with pollination experiments, aiming to provide pollen limitation values of animal-pollinated crops worldwide. Pollination experiments included hand pollen supplementation treatments, where plants were subjected to pollen supplementation of outcross pollen, and natural pollination treatments. The PolLimCrop dataset comprises 294 studies and 1169 unique pollen supplementation experiments with values of pollen limitation for 108 crops, spanning 50 years and 62 countries.

## Background & Summary

Pollination represents an important biodiversity-dependent ecosystem service for the provisioning of food and other human resources, with a significant impact on the global economy^1,2^. 75% of the leading worldwide crops depend on or, at least, benefit from insect pollination for marketable yield^3^, with increased cultivation of pollinator-dependent crops in the last decades^4,5^.

Animal pollinators are the main group responsible for pollen transportation between flowers, accounting for the pollination of crops that represent 35% of global food production^3^. When this transport is insufficient or inefficient, pollen deposition limitation is observed, which may result in lower fruit and/or seed quantity and/or quality^6,7^, and consequently, agricultural outputs may be affected, with associated economic losses^8,9^. When plants yield more fruits or seeds through hand pollen supplementation than from natural pollination, it indicates that production is likely constrained by pollen receipt. This difference in production between the two treatments can be used to calculate a pollen limitation (PL) value, which is often considered in the literature to reflect pollination service levels^10–12^. Inadequate pollination services are of particular concern given the current biodiversity loss^13^. Under scenarios of insect pollinator decline^14,15^ due to climate change, misuse of agrochemicals and anthropogenic changes in land cover and land management, which lead to landscape intensification and simplification^1^, it is urgent to identify the patterns and direct causes of pollen limitation in agroecosystems^12^. Given the global importance of animal pollinators, a compilation of crop pollination experiments is needed to identify productivity losses due to limited pollination services, which is vital to designing appropriate management practices and political frameworks for developing sustainable farming systems^5,16^.

**Fig. 1:**
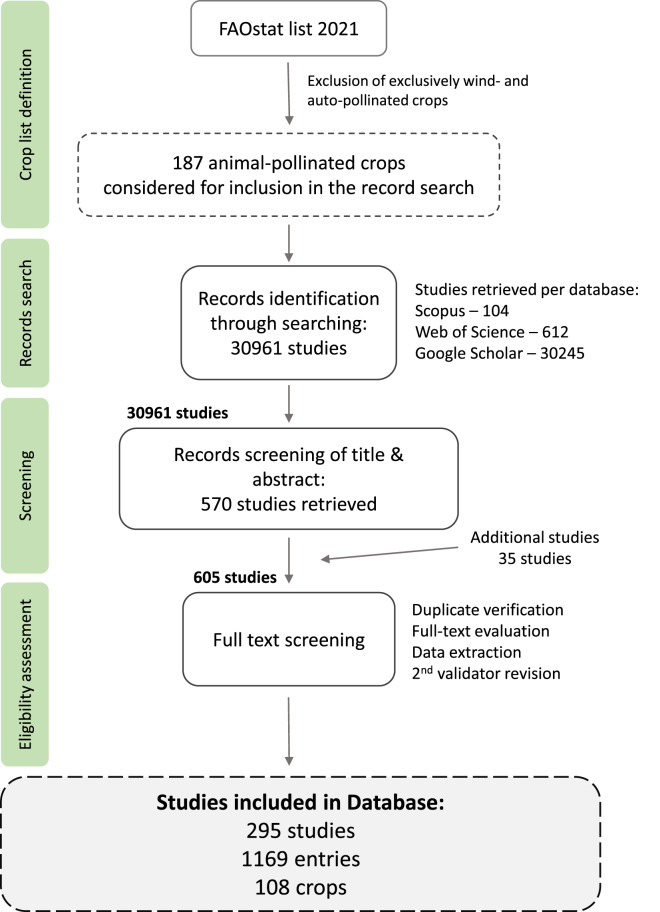
Detailed PRISMA flow diagram of conducted systematic search performed to compile the PolLimCrop dataset. Searches were performed on Scopus, Web of Science and Google Scholar (the first 1000 records were considered). Additional studies were obtained mainly through references and citations in the surveyed studies. These studies went through an equal screening as every other study. A second user revision was done by two validators who screened each study independently. Exclusion criteria from the 604 to the 294 studies are given in the dataset material^20^.

Here, we present the PolLimCrop dataset, which compiles data from 294 studies that performed hand pollination experiments, providing 1169 unique pollen supplementation experiments and PL values for 108 different animal-pollinated crops worldwide. PL is estimated from hand pollination supplementations with outcross pollen, considered here as the optimal pollination^10^ for pollen limitation calculation purposes, and natural pollination attained by local pollinator communities. Each data entry represents a unique pollination experiment with at least two treatments, the hand pollen supplementation treatment and natural pollination. In addition, important characteristics of the experimental design, such as crop accession (cultivars, varieties and other infraspecific taxonomic levels), hand pollen supplementation methodologies (i.e., H – hand pollen supplementation, BH – hand pollen supplementation with pollinator exclusion, EH – hand pollen supplementation with the emasculation of flowers, BEH – hand pollen supplementation with pollinator exclusion and the emasculation of flowers), level at which the hand pollen supplementation was applied (i.e., individual flower, branch, inflorescence, or entire plant), sample sizes and standard deviations, year and location (continent, country, locality and/or geographical coordinates) are also included, when available (all data descriptors are provided in Table 1). The workflow of PolLimCrop compilation is shown in Fig. 1.

**Table 1:** Descriptors included in the PolLimCrop dataset, with description and descriptor levels.

Descriptor name	Description	Descriptor levels
line	Unique identifier assigned to each line	Number [1–1169]
unicode	Unique code, constructed using “line”, the first 3 letters of the first author’s last name, “year of the experiment”, “crop”, “plant accession” and “factors”	NA
article code	Study identifier, represented by 1st author’s last name, publication year and DOI	e.g. Castro_2021_”DOI”
DOI citation	Study DOI or citation	NA
species	Species name of the crop	NA
crop name	Common name of the crop	NA
family	Plant family of the crop	NA
plant accession	Cultivar, subspecies, clone or another further taxonomic rank and/or subtype given by the published document	NA
crop part	Crop’s economically used part (i.e. seed or fruit)	Seed [S] or fruit [F]
continent	Continent location of the experiment	NA
country	Country location of the experiment	NA
locality	Specific location of the experiment	NA
latitude; longitude	Geographic coordinates (latitude, longitude) in decimal degrees. If not given, the most specific location was used for obtaining the coordinates	NA
precision	Precision indication for geographic coordinates	given coordinates [S]; estimated coordinates [E]
year of the experiment	Year in which the pollination experiment was performed (first year given in multiple-year experiments).	NA
scale	Scale levels of the experiment	Individual flower [flower], branch [branch], inflorescence or cluster [inflor.] or entire plant [plant]
supplement type	Additional treatments applied to the hand supplementation treatment	Hand pollen supplementation (HPS) [H], HPS with pollinator exclusion [BH], HPS with the emasculation of flowers [EH], HPS with pollinator exclusion and the emasculation of flowers [BEH]
factors	Attributes that make the entries statistically independent within the same study	NA
production variables + pollination treatments + type of data	Data (mean [m], standard deviation [sd], and sample number [n]) related to production variables associated with the pollination treatments included in the dataset	Production variable levels: Fruit set [FS], seed set [SS], seed number [SN], fruit weight [FW] and seed weight [SW]; Pollination treatment levels: hand pollen supplementation [SUP], natural pollination [NAT], pollinator exclusion [BAG]; e.g. FS_SUP_m
PL proportion	Pollen deposition limitation (PL) proportion values calculated for each entry	In proportion (0–1)
PL effect size	Pollen deposition limitation (PL) effect size values calculated for each entry	Effect size (log response ratio)
effect size constant	Constant use indication for calculation of the effect size	With constant [Y]; with no constant [N]

The PolLimCrop dataset allows the assessment of trends in yield losses due to insufficient or ineffective crop pollination services, and represents a valuable resource for researchers, policymakers and related practitioners. One publication has so far resulted from PolLimCrop, focused on studying methodological aspects regarding pollinator dependence calculation and with a quantitative compilation of pollinator dependence values for crops^17^, but many topics remain to be explored. Understanding these trends can identify and inform necessary agricultural management practice changes to improve agroecosystem pollination services.

## Methods

### Literature search and data extraction

We gathered publications that reported pollination experiments in animal-pollinated crops through a systematic review using the following three databases: Web of Science, Scopus and Google Scholar. We based our search on a list of animal-pollinated crops (based on the list of produced crops of FAO 2021^18^). Our search did not include crops known to be exclusively wind-pollinated or reproduce exclusively through auto-pollination. We focused on pollination experiments performed in agricultural contexts, which had the assessment of production levels after at least two treatments: 1) natural pollination, where plants were exposed to natural pollination services present in the study region, and 2) hand supplementation, where plants were subjected to hand pollen supplementation of outcrossing pollen.

The search was conducted in two steps and used search terms for publications from January 1^st^, 1900, to March 1^st^, 2022. First, we conducted a general search (performed on Web of Science, Scopus and Google Scholar) with the string “Crop” AND (“hand” OR “suppl”) AND (“natural” OR “open”) AND “pollination”, and the string “Crop” AND “hand” AND (“pollen application” OR “supplementation”) AND (“natural” OR “open”) AND (“fruit” OR “seed”) AND “pollination” NOT “wild plant community” NOT “natural pop”. Second, we searched for each selected crop (performed on Web of Science and Google Scholar) with the string “species name” AND (“hand” OR “suppl”) AND (“natural” OR “open”) AND “pollination”, and the string “crop common name” AND (“hand” OR “suppl”) AND (“natural” OR “open”) AND “pollination”. All available publication formats were considered (e.g. published article, poster, thesis, report), verifying for duplicated data among the different formats to avoid duplicates. The literature search, selection process and exclusion criteria are illustrated in a PRISMA flow diagram (Fig. 1).

The 30,961 records retrieved from the three above-mentioned databases were sieved through a first eligibility screening based on title and abstract reading. All records likely having pollination experiments passed to the second phase of analyses, resulting in 604 studies. Then, these studies were carefully evaluated through full-text reading and review, retrieving data for the dataset from 294 records. The details on the acceptance/rejection decision for each of the 604 studies are provided in the dataset material. A data entry in the dataset consisted of a unique pollination experiment made in a given crop (and accession) in a specific location and season. For each data entry, we collected crop production values for each pollination treatment, including the following response variables (when available): fruit set, fruit weight, seed set, seed weight, and/or seed number. We report the mean value of the given response variable for each pollination treatment and the standard deviation (SD) and sample size whenever provided. When standard deviation was not provided, it was obtained from other variables (e.g., standard error), whenever possible. In binomial variables, such as fruit and seed sets, if the standard deviation was not provided, it was estimated from the mean and sample size^19^. Whenever data was given in the graphical form, we extracted the values using ImageJ (version 1.53r April 21^st^, 2022). Also, information on pollinator exclusion treatment (i.e., the bagged treatment) was collected when available. We also extracted geographical information, i.e., continent, country and city and geographical coordinates of the pollination experiment, and year of the experiment, crop family, species, common name and accession (i.e., cultivars, varieties and other infraspecific taxonomic levels), part of the crop economically used (i.e., fruit, seeds or both) and methodological details related with pollination experiments, i.e., additional treatments of the hand supplementation (i.e., hand pollen supplementation, only; pollinator exclusion and hand pollen supplementation; emasculation and hand pollen supplementation; pollinator exclusion, emasculation and hand pollen supplementation), and scale of the experiment (i.e., single flower, branch, inflorescence/cluster, or entire plant). All extracted descriptors are provided in detail in Table 1.

### Pollen limitation calculation

Pollen limitation (PL) value was calculated as a PL ratio for each entry using the following equation^20^:

$$PL(proportion)=1-\frac{natural\,pollination}{hand\,pollination}$$

Natural pollination represents the plant reproductive success after natural levels of pollination services in a given experimental location and time, and hand pollination represents the plant reproductive success after hand pollen supplementation treatment. The index was estimated for each entry, using the available production variables, depending on the part of the crop used economically (i.e., fruit, seeds or both). In fruit crops, fruit-related variables were used, namely fruit set and weight. Similarly, seed-related variables were used for PL estimation in seed crops, i.e., fruit set, seed set, seed number, and seed weight. In crops where both parts are economically used, all available variables were used to calculate PL. When more than one production variable was present, a mean value of PL using the available variables (given in column [PL <used variable>]) was calculated. When production following natural pollination was equal to or higher than the hand pollen supplementation, pollen limitation was considered 0. This way, PL varied from 0 (i.e., absence of pollen limitation) to 1 (maximum pollen limitation). The PL value for each entry is provided in the PL_proportion column of the dataset.

Additionally, the magnitude of PL effect was calculated as the log response ratio for each entry using the following equation^7^:

$$PL\left(effect\;size\right)=ln\left(\frac{hand\;pollination}{natural\;pollination}\right)$$

Following the above-mentioned methodology, each entry’s effect size was estimated using the available production variables. However, the log response ratio does not compute estimates when zero events occur. This occurred 29 times for the fruit set and four times for the seed number (no zero events were detected in the remaining production variables). Although adding a constant to zero events is generally not recommended in the literature, not estimating the effect size for these entries (which mainly occurred on the natural treatment side, indicating strong pollen limitation) may lead to underestimations of PL. Also, adding a constant to these entries did not produce high effect size values in relation to the overall dataset. Thus, a constant was added to both treatments for entries with zero events. Namely, a constant of 1 was added for the fruit set variable, and a constant of 0.001 was added for the seed number variable. The entries where the constant was applied to calculate effect size are indicated in the dataset (in the column effect_size_constant). Effect size assumed negative and positive values, with positive values indicating pollen limitation and negative and 0 values indicating the absence of pollen limitation. The PL effect size for each entry is provided in the PL_effectsize column of the dataset. However, because the dataset provides raw data for each pollination treatment, future studies can select specific response variables and explore other ways to calculate PL values.

### Imagery and Maps construction

ArcGIS (version ArcGIS Pro 3.0.3) was used to map all entries using the study location of each dataset entry (provided in the dataset). The extracted geographical information included continent, country, city or locality and geographical coordinates (given as latitude and longitude) of the pollination experiment. When geographical description did not include geographic coordinates, approximated coordinates were calculated through Google Earth images using the most precise given location possible, i.e., if a city or locality was given, the geographical coordinates of the city centre were considered. The dataset includes information on the geographical coordinates source. Finally, R was used for obtaining the remaining graphs and images (R version 4.2.1) through the package ‘ggplot2’.

### Dataset characteristics

**Fig. 2:**
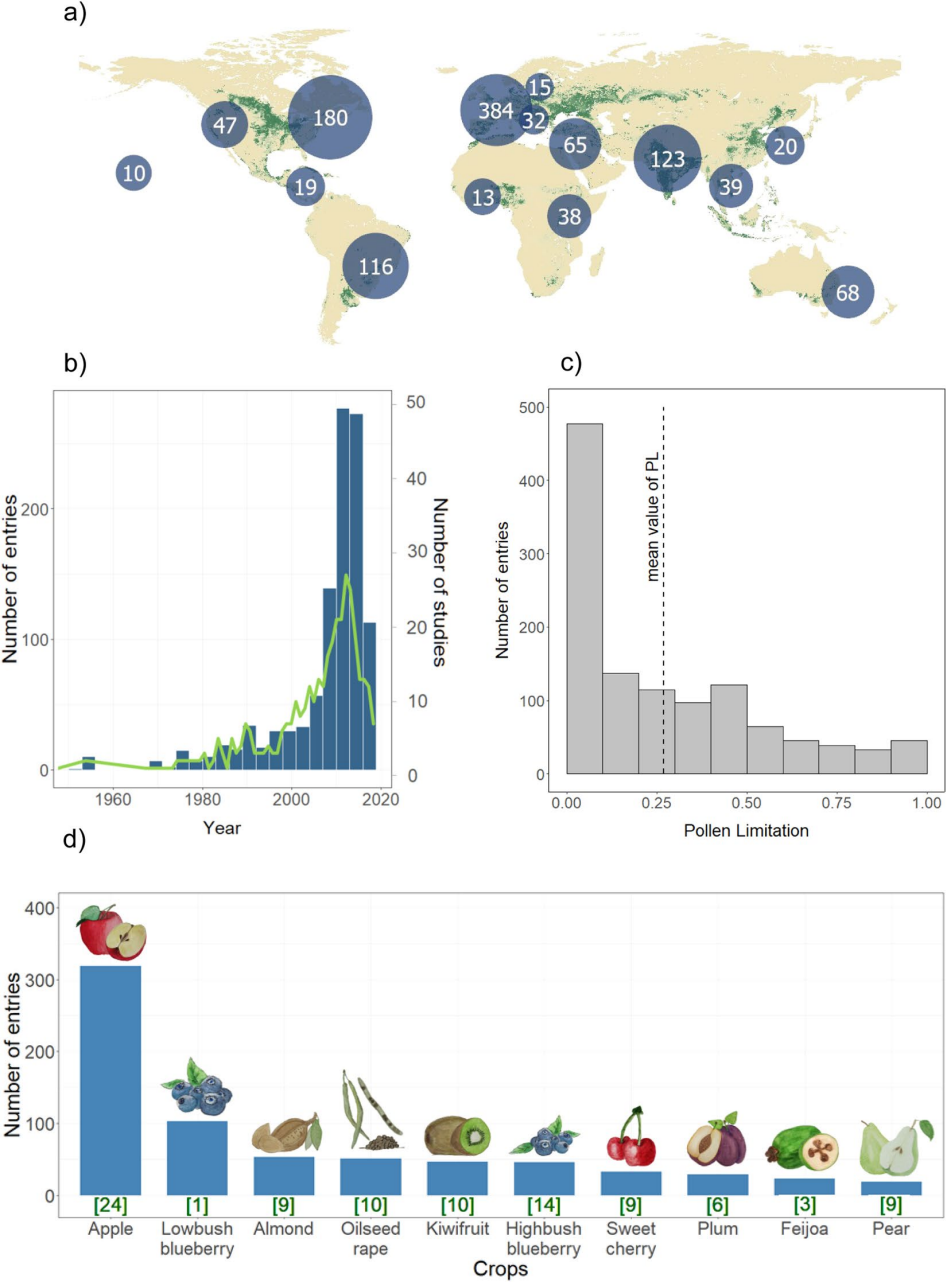
(**a**) Global distribution of data points of the PolLimCrop dataset; blue circles size represent the total number of entries for the different regions; green areas represent cropland areas in 2020^25^. (**b**) Total number of entries (indicated with blue bars; left axis) of the PolLimCrop dataset and the total number of studies (indicated with a green line; right axis) along the years (from 1950 to 2020). (**c**) Distribution of data points based on pollen limitation values; values are given in proportion (0 represents no pollen limitation, and 1 represents maximum pollen limitation); the dashed line indicates the overall mean value of pollen limitation of the animal-pollinated crops included in the PolLimCrop dataset. (**d**) Number of entries for the 10 animal-pollinated crops with the highest representation in the studies included in the PolLimCrop dataset (blue bars), with the total number of studies per crop being indicated below each bar (in square brackets).

The PolLimCrop dataset includes data from experiments done in 62 countries and 5 continents, covering the major crop production regions in the world (green areas in Fig. 2a). Europe, North America, and Asia are the most represented regions, with 36.9%, 21.5% and 20.6% of the entries, respectively (Fig. 2a). The available studies provide data on pollination experiments performed since 1950; still, most data come from the 21^st^ century (representing 77% of total entries; Fig. 2b). Although the number of studies has increased in the last two decades, we still lack information for many crops and locations where the crop is grown, and the information on PL available for crops is far more reduced than for wild plants^7^.

Overall, an average increase of 27% in production was observed after a pollen supplementation treatment. PL values, given as proportion in the dataset, span from no pollen limitation (PL = 0), where pollen supplementation does not lead to a production increase, to maximum pollen limitation (PL = 1), where pollen supplementation leads to a production increase of 100% compared with the natural levels of pollination (Fig. 2c). The crops that contributed most to the number of entries are represented in Fig. 2d,with apple representing 27.2% of the entries, followed by lowbush blueberry (8.8%), almond (4.5%) and oilseed rape (4.4%).

## Data Records

The PolLimCrop dataset is available for download at figshare^21^. The dataset file includes 5 parts: 1.csv file and 4.txt files. The primary dataset file, “PolLimCrop_dataset”, contains one sheet organized by line number. There are 1169 records, with data from 294 studies and 108 crops. The dataset includes 66 columns: “line”, “record_unicode”, “article_code”, “DOI_citation”, “species”, “crop_name”, “family”, “plant_acession”, “crop_part”, “continent”, “country”, “locality”, “latitude;longitude”, “precision” “experiment_year”, “scale”, “supplement_type”, “factors”, and 45 columns with data of the included production variables, i.e., fruit set (FS), seed set (SS), seed number (SN), fruit weight (FW) and seed weight (SW), with mean, standard deviation (SD) and sample number data provided for each of the pollination treatments, i.e., supplemental pollination (SUP), observed pollination (OBS) and pollinator exclusion (BAG). Lastly, the dataset includes two final columns with estimated PL, “PL_proportion” and “PL_effect_size”, using entry data available and the column “effect_size_constant”. The file “PL_Calculation_Variation_extraction” contains the PL estimation process originating column “PL” and information on the extraction of standard deviation data. Detailed information explaining each column is provided in the file “Column_Descriptor” and Table 1.

## Technical Validation

### Dataset validation

All entries in the dataset were validated by a second person against the original source, and any record with inconsistencies was discussed among all validators. Plant species were confirmed to follow the currently accepted taxonomy, according to The World Flora Online (available at: http://www.worldfloraonline.org). In the cases where the published study used a synonym of an accepted species name, we provide the accepted species name.

### Dataset limitations and discussion

Some limitations of the PolLimCrop dataset need to be considered in future studies. First, the search criteria used the English language, and thus, it excluded studies published in languages other than English, although articles written in such languages but with an abstract in English were revised and included. Consequently, the dataset may have a language bias in the selection of the studies, which might reduce the number of studies from certain regions of the world. This may partially explain the high representation of studies from Europe and North America. Such limitations should be considered in future analyses that use the PolLimCrop dataset, as they may lead to bias in result analyses^22^.

Second, for 285 entries, hand pollen supplementation resulted in lower production levels than open pollination. This could be explained by the fact that, for certain circumstances, applying large loads of pollen can decrease reproductive success due to pollen clogging and/or pollen competition^23,24^. Alternatively, methodological errors may also have contributed to such outcomes; among methodological problems, there could be the use of low-quality or unviable pollen, limited genetic diversity due to the use of a low number of pollen donors, or damage to the reproductive units during the hand pollination experiments^23,24^. These outcomes may impact the dataset, potentially underestimating the overall PL ratio.

### Supplementary information

PolLimCrop_dataset

## Data Availability

The codes used to produce figures in this manuscript (Fig. 2b–d) are available in R programming language on the main GitHub repository: https://github.com/catarinasiopa/PolLimCrop.git.
